# Characterization and Clinical Relevance of Endometrial CAFs: Correlation between Post-Surgery Event and Resistance to Drugs

**DOI:** 10.3390/ijms24076449

**Published:** 2023-03-29

**Authors:** Raed Sulaiman, Pradip De, Jennifer C. Aske, Xiaoqian Lin, Adam Dale, Kris Gaster, Luis Rojas Espaillat, David Starks, Nandini Dey

**Affiliations:** 1 Department of Pathology, Avera Research Institute, Sioux Falls, SD 57105, USA; 2 Translational Oncology Laboratory, Avera Research Institute, Sioux Falls, SD 57105, USA; 3 Department of Internal Medicine, University of South Dakota SSOM, USD, Sioux Falls, SD 57105, USA; 4 Viecure, Greenwood Village, CO 80111, USA; 5 Assistant VP Outpatient Cancer Clinics, Avera Cancer Institute, Sioux Falls, SD 57105, USA; 6 Department of Gynecologic Oncology, Avera Cancer Institute, Sioux Falls, SD 57105, USA

**Keywords:** CAFs primary culture, CAF markers, patient-specific aggressive CAFs, post-surgery event, drug resistance, endometrial cancers

## Abstract

Cancer-associated fibroblasts (CAFs) within a solid tumor can support the progression of cancer. We studied the identification and characterization of patient-derived endometrial CAFs in the context of their clinical relevance in endometrial cancers. We established patient-derived primary cultures of CAFs from surgically resected tumors (TCAF) and tumor-adjacent normal (NCAF) tissues in 53 consented patients with success rates of 97.7% and 75%, respectively. A passage of CAF was qualified by the (1) absence of CK 8,18,19, EpCAM, CD45, and CD31, and (2) presence of SMAalpha, S100A4, CD90, FAP, TE-7, CD155, PD-L1, TGFB, PDGFRA (qRT-PCR, flow cytometry, Western blot, ICC). Out of the 44 established CAFs, 31 were aggressive (having an early, i.e., 4–7 week, establishment time and/or >3 passages) compared to 13 which were non-aggressive. A post-surgery-event (PSE) was observed in 7 out of 31 patients bearing aggressive CAFs, 2 of whom were also positive for CTCs, while none of the 13 patients bearing non-aggressive CAFs had events. A positive correlation was found between patients with grade 3 (*p* = 0.025) as well as stage 3/4 diseases (*p* = 0.0106) bearing aggressive CAFs and the PSE. Finally, aggressive TCAFs from patients with PSE resisted the effects of paclitaxel and lenvatinib on the growth of HUVEC and endometrial tumor cells. Our study is the first to report *a correlation between the PSE and the aggressive nature of CAFs in endometrial cancers* and provides an undeniable reason to study the in-depth mechanism of CAF function towards the development of treatment resistance in endometrial cancers.

## 1. Introduction

### Statement of Translational Relevance

Endometrial cancer-associated fibroblasts (CAFs) can be designated as aggressive or non-aggressive within 4–7 weeks of the time of surgery based on the characteristics of the established primary ex vivo culture. An aggressive CAF from an individual patient with a high grade/stage of the disease can be tested for its role in the development of resistance to the same drugs (chemotherapy, targeted, and/or anti-angiogenic drugs) received as adjuvant treatment within eight weeks of the surgery. The designation of the aggressiveness of an established CAF will provide an opportunity for a personalized test to predict the development of a TME/CAF-mediated resistance to the adjuvant treatment in real-time, prior to clinically recorded events. Thus, the data obtained from our study present a not-so-far-reported *mode of personalized testing for the development of real-time resistance to adjuvant therapy in high-risk patients* with endometrial cancers well before it is clinically encountered in the patients.

Endometrial cancer is the most common gynecological malignancy in high-income countries, and the highest rate of this cancer is currently observed in North America [1,2]. The incidence in the United States in recent years is making uterine cancer the fourth most common cancer in women and the fifth most common cause of cancer death, and its five-year, age-adjusted survival has not improved [3]. With high-grade endometrial cancers that tend to recur, it is desirable to prevent recurrence as the prognosis for recurrence is dismal [1]. Extra-pelvic disease recurrence/recurred metastatic endometrial cancers have a limited response to standard therapies, and the overall survival for most patients with recurrent or metastatic disease is around one year [4,5], thus highlighting an unmet need for a novel treatment strategy.

Tumor cells within a progressive disease enforce several pro-metastatic event(s) via direct and indirect cross-talk with the immune, angiogenic, and mesenchymal stromal cells of TME. CAFs, the most abundant and influential cells of TME, have a significant contextual role in this progression by means of resisting the therapy and ensuing metastasis reported in colon, pancreas, breast, esophagus, and skin cancers. Understanding CAFs’ undeniable role in the resistance to therapy and the progression of solid tumors has recently encouraged CAF-directed clinical trials as part of a next-generation cancer drug design and discovery innovation strategy [6,7,8,9].

Acknowledging the fact that CAFs partner with tumor cells and all components of TME in a solid tumor, working in favor of the progression in spite of therapy [8], and the specific role of endometrial CAFs in the disease progression [7], we hypothesize that endometrial tumors bear characteristic CAFs which have a clinical relevance from the viewpoint of post-surgery events (PSE). To test our hypothesis, we generated patient-derived CAFs from resected tumor tissues (TCAF) and tumor-adjacent normal tissues (NCAF) at the time of surgery. We identified aggressive CAFs as defined by an early establishment (within 4–7 weeks) and a higher passage number (>3 passages) of the primary culture. By designating the aggressiveness of the CAFs from each patient’s tumor sample, we *tested the clinical relevance of the aggressiveness of CAFs and demonstrated that the post-surgery event in patients with a high grade and stage of the disease is directly correlated to the aggressive nature of the CAFs in endometrial cancers.* Aggressive endometrial CAFs from patients with PSE resisted the effects of paclitaxel and lenvatinib on the growth of endometrial cells and HUVEC cells in a hybrid-co-culture (HyCC), indicating an effect mediated via secretomic paracrine signals as compared to direct contact.

## 2. Results

Our cohort involved 53 patients with endometrial cancers who provided informed consent for the study. Table 1 presents information about the patients included in the study. CAFs were established from both T and TAN received at the time of surgery, depending on the availability of tissue samples. Out of 53 tissue samples, 9 samples were used for the standardization of the CAFs. A total of 44 tissue samples from patients were used for establishing CAF cultures. Out of 44 patient samples, 39 TCAFs (5 failed to grow) and 15 NCAFs (4 failed to grow) were established. In many instances, we received tumor tissue paired with tumor-adjacent normal tissue or Fallopian tubes as the normal tissue.

As we progressed from getting consent from the patients to characterizing the primary CAFs for our study, we passed through different steps, culminating in different CAF samples for the study. Sometimes, we received unpaired samples. Sometimes, we received Fallopian tube samples as the tumor-adjacent normal tissue from the pathology. In our experience, none of the Fallopian tube samples produced NCAFs in our way of creating a non-enzymatic culture from a feeder layer. Often, the paired samples did not grow equally to yield CAFs for passages and characterization by ICC, qRT-PCR, WB, and flow cytometry, which led to the differences in the number of CAF samples in the study.

### 2.1. Characterization of Patient-Derived Endometrial CAFs

**Figure 1:**
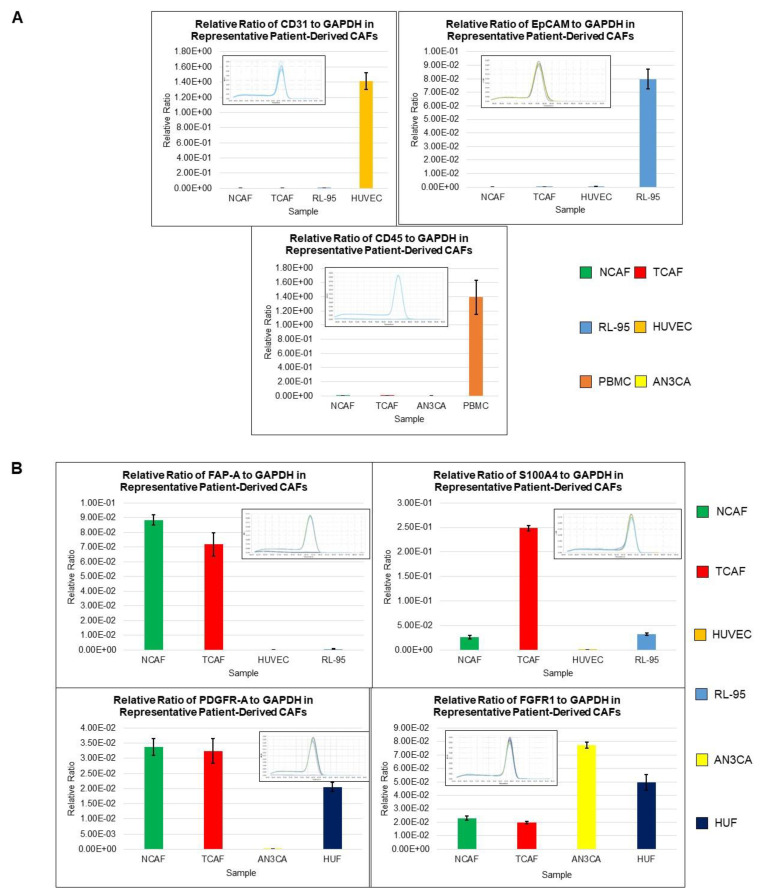

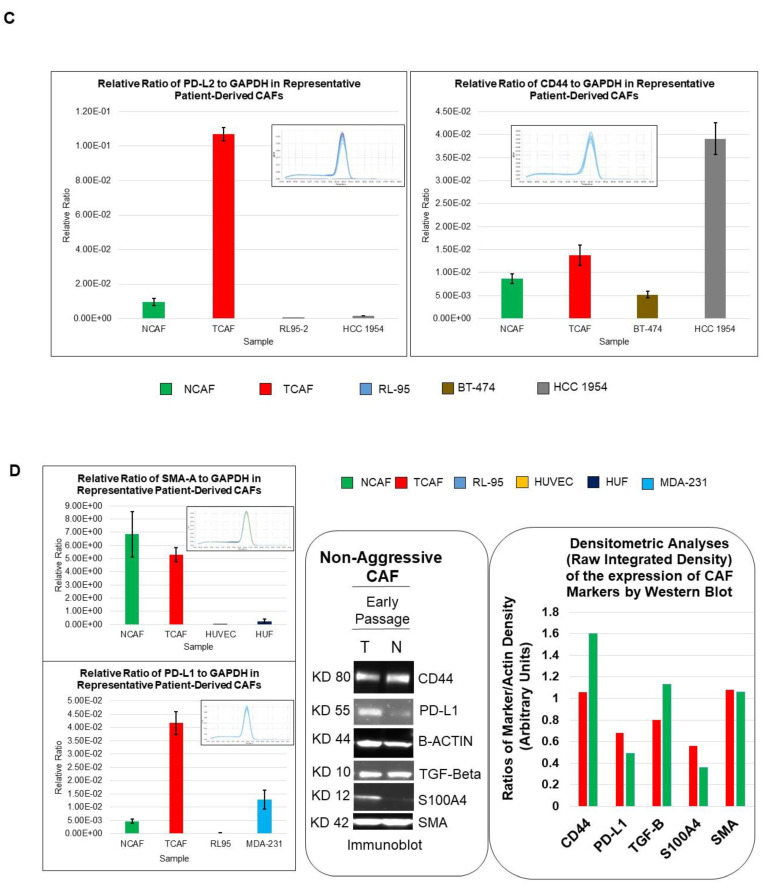
Expression of mRNA for marker proteins of CAF by qRT-PCR: Each graph represents the expression of a gene of interest presented as its relative ratio to *GAPDH*. Inset in each graph is a melting curve of the gene of interest. CAF samples and the positive and negative controls for each gene are color-coded and listed at the bottom of the figure. (**A**) presents expressions of three negative markers of CAF, endothelial marker (*CD31*), epithelial marker (*EpCAM*), and common leucocyte antigen (*CD45*), in NCAFs and TCAFs with respective validation controls. (**B**) presents expressions of four positive markers of CAF (*FAP-A*, *S100A4, PDGFR-A*, and *FGFR-1*) in NCAFs and TCAFs with respective validation controls. (**C**) presents expressions of *PD-L2* and *CD44* in NCAFs and TCAFs with respective validation controls. (**D**) compares the qRT-PCR expression of markers with the protein expression of the same markers (SMA and PD-L1) by WB (inset) from a representative non-aggressive early passage NCAF and TCAF. The expressions of CD44, TGFB, and S100A4 by WB are also presented with the corresponding densitometric analyses (bar diagrams) using Image J. Beta-actin was used as the loading control.

**Figure 2:**
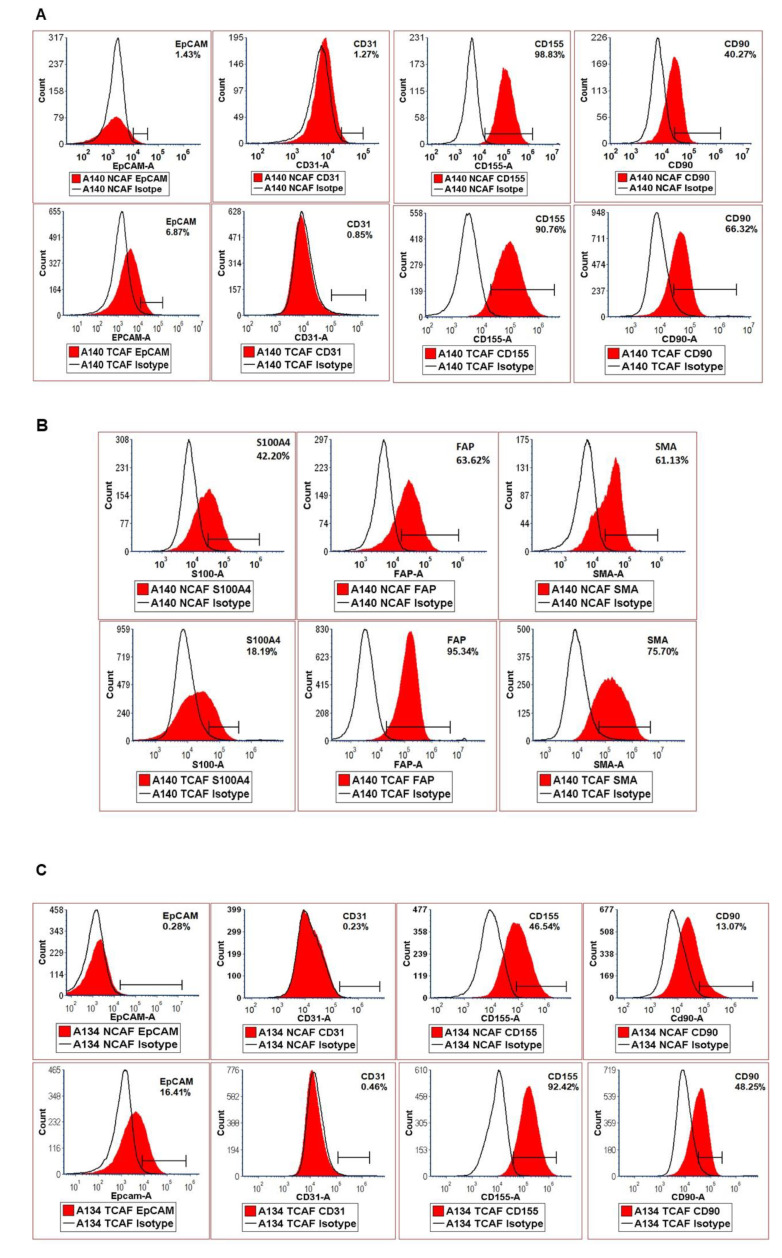

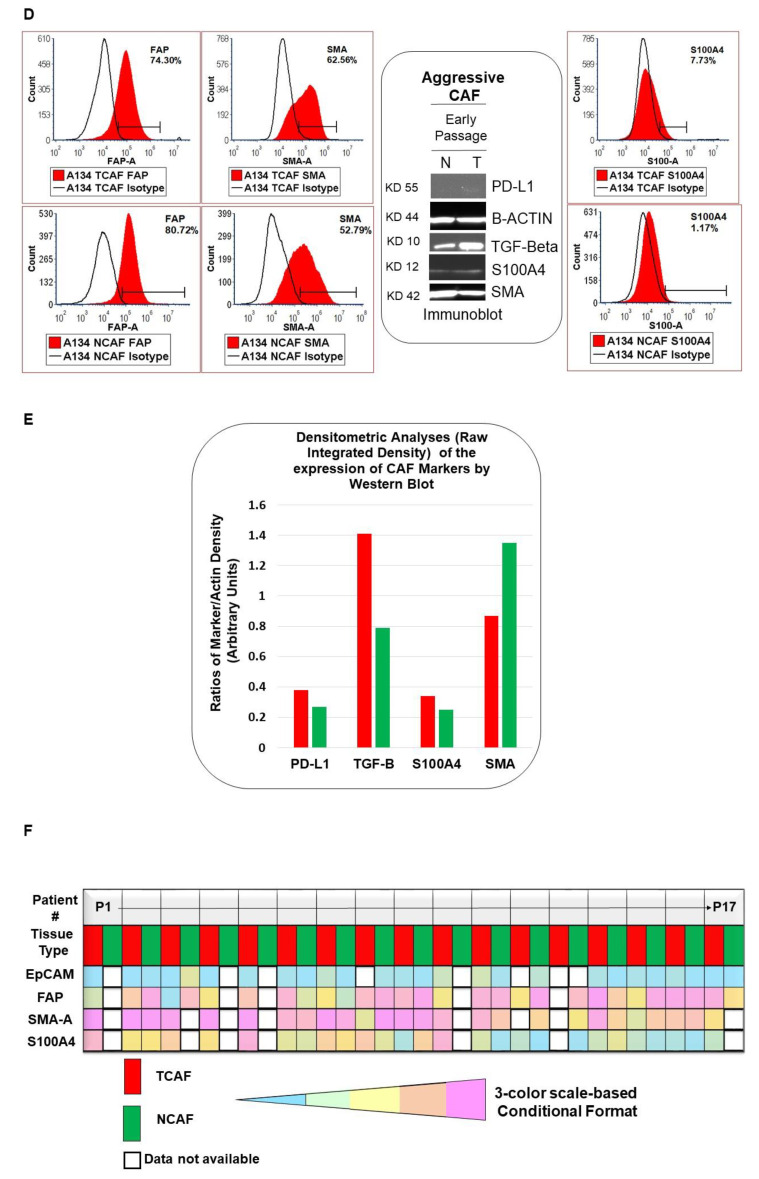
Expression of marker proteins of CAF by flow cytometry: Expression of the six positives (CD155, CD90, SMA-A, S100A4, FAP-A) and two negatives (EpCAM and CD31) markers of CAF in the established TCAFs and NCAFs from the T and TAN samples from two patients; one bearing non-aggressive CAFs (**A**,**B**) and bearing aggressive CAFs (**C**,**D**) by flow cytometry. (**D**) compares the flow cytometry expression of SMA and S100A with the WB expression (inset) of SMA and S100A from the same representative aggressive CAFs (TCAF and NCAF). The expressions of PD-L1, and TGFB, by WB, are also presented. The protein expression of the same markers (SMA and S100A4) by WB (inset) from a representative aggressive early passage NCAF and TCAF. The expressions of other CAF markers, such as PD-L1 and TGFB, by WB are also presented with the corresponding densitometric analyses (bar diagrams) using Image J (**E**). Beta-actin was used as the loading control. The isotype controls for the respective protein are marked as open black lines compared to the red area under curves for the proteins. A three-color scale-based conditional formatting of the pattern of % expression of CAF markers of EpCAM, SMA-A, S100A4, and FAP (by flow cytometry) in the pairs of cultured TCAFs and NCAFs (early passages) from 17 representative patients (as presented with color codes) is presented as a heatmap (**F**). Red bars represent TCAFs and green bars represent NCAFs (**E**).

**Figure 3:**
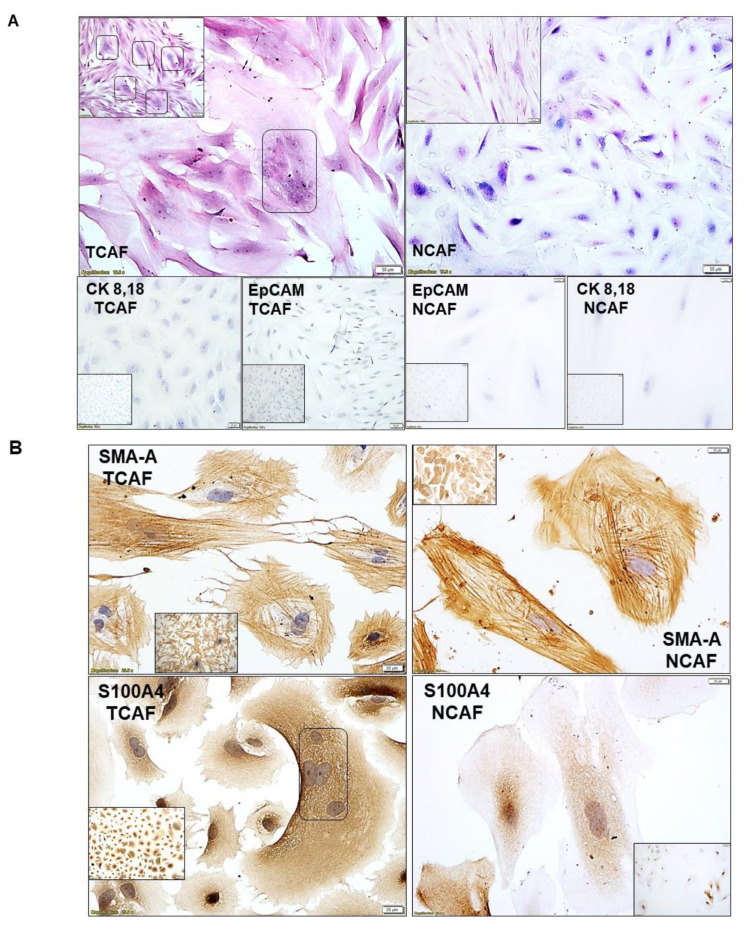

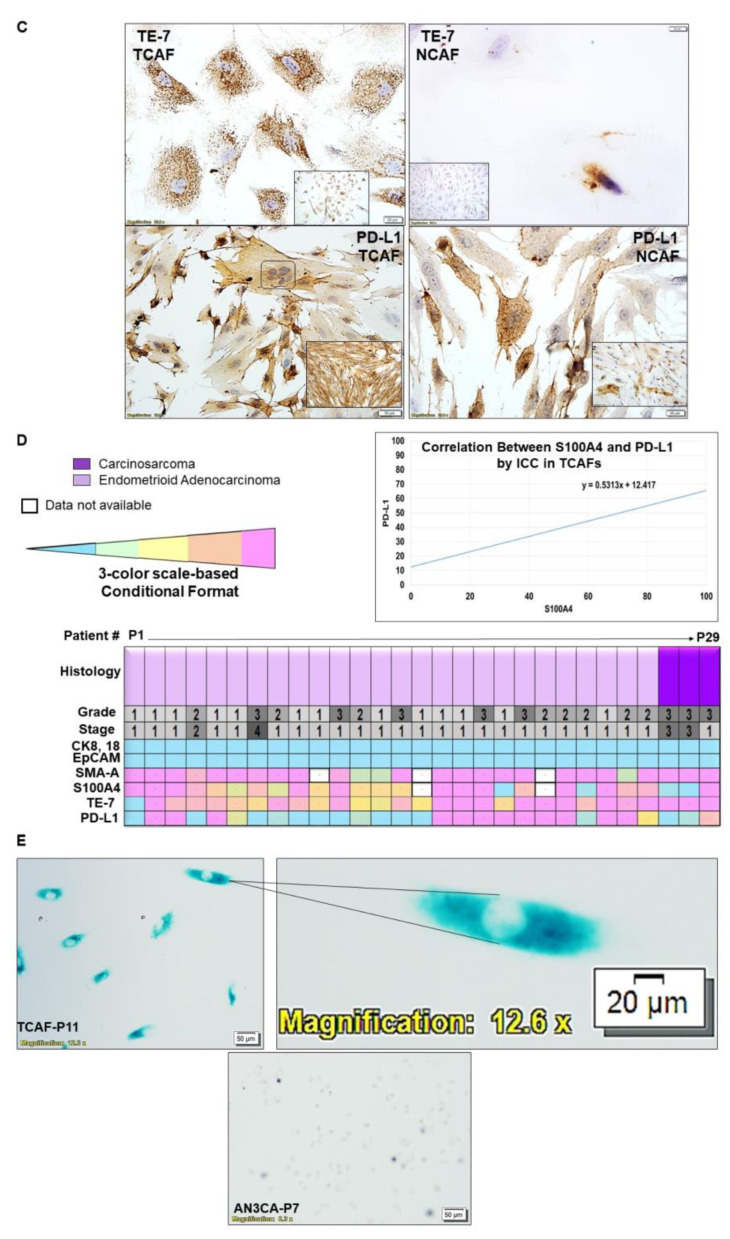
Representative photomicrographs showing subcellular localization of different positive markers of CAF, SMAalpha, S100A4, TE-7, and PD-L1, as well as negative markers, EpCAM and CK 8,18 of CAFs in established primary cultures of NCAF and TCAF samples from patients with endometrial cancers by ICC: Images show H&E (**A**) upper panel, and negative markers such as CK 8,18 and EpCAM (**A**) lower panel as well as expressions of positive markers including SMAalpha (**B**) upper panel, S100A (**B**) lower panel, TE-7 (**C**) upper panel, and PD-L1 (**C**) lower panel in NCAFs and TCAFs of tumor samples from different patients. Hematoxylin was used as the counterstain. For ICC, pictures were taken at 20× and 40× dry-objective of Olympus BX43 Microscope using cellSens 1.18 LIFE SCIENCE IMAGING SOFTWARE (OLYMPUS CORPORATION). Photomicrographs show the information stamps for magnifications and the scale bars. A three-color scale-based conditional formatting of the pattern of % expression of CAF markers of CK 8,18, EpCAM, SMAalpha, S100A4, and TE-7 (by ICC) in cultured TCAFs (early passages) from 29 representative patients sorted on the basis of histology of the disease with corresponding grades/stages (as presented with color codes and numbers) is presented as a heatmap (**D**). The heatmap displays the % expression of CK 8,18, EpCAM, SMAalpha, S100A4, and TE-7 in TCAFs from 29 out of a total of 31 patients as evaluated by ICC. A very similar pattern of the expression of markers in TCAFs between different histologies, endometrioid adenocarcinoma, and carcinosarcomas of endometrial cancers was observed, although the number of patients for the latter was much smaller in the cohort. Inset shows a positive correlation between the % expressions of S100A4 and PD-L1 in TCAFs in these 27 patients out of a total of 29 patients. Trendline demonstrated the Pearson correlation between S100A4 and PD-L1 by ICC in TCAFs from patients. *p* = 0.0373, *n* = 27. Created using GraphPad Prism Version 9.4.1. The beta-galactosidase stain of endometrial CAFs of late passage (P11) as compared to epithelial tumor cell lines of endometrial AN3CA cell line (P7): Endometrial CAFs stain positive in contrast to AN3CA endometrial cancer cell line (**E**). Inset shows a higher magnification of CAF (the scale bar is computer-generated), showing the subcellular distribution of the stain.

The purity and specificity of the CAFs were tested by (1) the negative expression of non-CAF markers, including CK 8,18,19, EpCAM, CD45, and CD31, and (2) the positive expression of SMAalpha, S100A4, CD90, FAP, TE-7, TGFB, FGFR1, and PDGFRA. The CAFs were characterized by testing the expression of mRNA using qRT-PCR as well as protein expression using flow cytometry and ICC. The CAFs established from the patient samples were characterized by a negative expression of cell type markers, including CD45, CD31, EpCAM, and CK 8,18, with a parallel positive expression of SMAalpha, FAP, S100A4, and TE-7. PD-L1 and CD44 were additionally used separately. The success rates of the establishment of CAFs were 97.7% (43 out of 44) and 80% (12 out of 15) for TCAFs and NCAFs, respectively. Figure 1A–D presents the expressions of mRNA for different positive (*SMA, FAP-A, S100A4, PDGFR-A, FGFR1, CD44, PD-L1, PD-L2*) and negative (*CD31, EpCAM, CD45*) markers of CAF in endometrial NCAFs and TCAFs as obtained using qRT-PCR. Figure 1D compares the qRT-PCR expression of certain markers with the protein expression of the same markers and more using the WB of a representative non-aggressive NCAF and TCAF pair, demonstrating the expression of SMA in both in contrast to a higher expression of PD-L1 in TCAF than in NCAF. Figure 2 presents the expression of the protein markers of CAF in endometrial NCAF and TCAF pairs using flow cytometry. It depicts the expression of the six positive (CD155, CD90, SMA-A, PD-L1, S100A4, FAP-A) and two negative (EpCAM and CD31) markers of CAF in the established aggressive TCAFs and NCAFs from the tumor samples of two patients (A, B and C, D) with endometrial cancers by flow cytometry. Although the expressions in TCAFs and NCAFs were found to be comparable in terms of both negative and positive markers, a general trend of a higher expression of FAP and CD90 was noted in TCAFs. Figure 2D demonstrates a flow cytometry expression of SMA and S100A4 comparable to the immunoblot expression from representative aggressive CAFs (TCAF and NCAF). The immunoblot expressions of PD-L1 and TGFB are also presented (Figure 2E; densitometric analyses from WB by Image J). A three-color scale-based conditional formatting of the pattern of the % expression of CAF markers of EpCAM, SMA-A, S100A4, and FAP in the pairs of cultured TCAFs and NCAFs (early passages) from 17 representative patients (as presented with color codes) is presented as a heatmap (Figure 2F) which shows a comparable pattern of % distribution of the markers in NCAFs and TCAFs. Figure 3 presents the representative photomicrographs (Figure 3A–C) of the subcellular expression of different positive (CAF-specific markers such as SMA, S100A4, and TE-7, and immune checkpoint marker PD-L1) and negative markers (CK 8,18,19 and EpCAM) of CAF obtained using ICC. The NCAFs and TCAFs are indistinguishable morphologically by H&E stain, although their expression patterns vary for marker proteins. We observed multinucleated TCAFs in a few patients’ tumor samples (Figure 3A upper panel left). Figure 3B,C presents a representative photomicrograph of a positive expression of SMAalpha, S100A4, TE-7, and PD-L1 in the NCAFs and TCAFs of different patients, respectively. Noticeably, SMAalpha, S100A4, and PD-L1 were expressed in multinucleated NCAFs and TCAFs. Both epithelial markers, CK 8,18,19 and EpCAM, were negative in all TCAFs and NCAFs (Figure 2A lower panel). A heatmap of the % expression (Figure 3D) of the positive and negative marker proteins of TCAFs from 29 patients with endometrial cancers of different histologies, stages, and grades obtained using ICC is presented in Figure 3D. The heatmap of the expression of the markers in the TCAFs showed (1) a uniformity of the negative expression of the epithelial markers CK 8,18,19 and EpCAM in all TCAFs, (2) a uniformity of the expression of the fibroblast markers SMAalpha and TE-7 in all TCAFs, and (3) a differential expression of S100A4 and PD-L1 in TCAFs irrespective of the histologies, grades, and stages of the tumors. The inset shows a positive correlation between PD-L1 positive TCAFs and S100A4 positive TCAFs in 27 patients. Trendline demonstrated the Pearson correlation between S100A4 and PD-L1 as obtained by ICC in TCAFs from patients (*p* = 0.0373, *n* = 27; Created using GraphPad Prism Version 9.4.0).

### 2.2. Designation of Aggressive and Non-Aggressive Endometrial CAFs

We categorized CAFs based on criteria concerning their growth patterns, as mentioned in the method section. Out of established patient-derived CAFs, we obtained aggressive CAFs from 31 patients and non-aggressive CAFs from 13 patients. Interestingly, all 31 patients in the aggressive CAF category had TCAFs. We identified six patients with aggressive NCAFs. Two patients had aggressive TCAFs but non-aggressive NCAFs. Two out of seven aggressive CAF-bearing patients’ blood was positive for CTC, as shown using a laboratory-friendly method of double-immunocytochemistry and triple-immunofluorescence as detailed elsewhere [10]. Table 2 presents the patients’ information for the aggressive and non-aggressive CAFs.

### 2.3. Clinical Relevance of Patient-Derived Aggressive Endometrial CAFs

**Figure 4:**
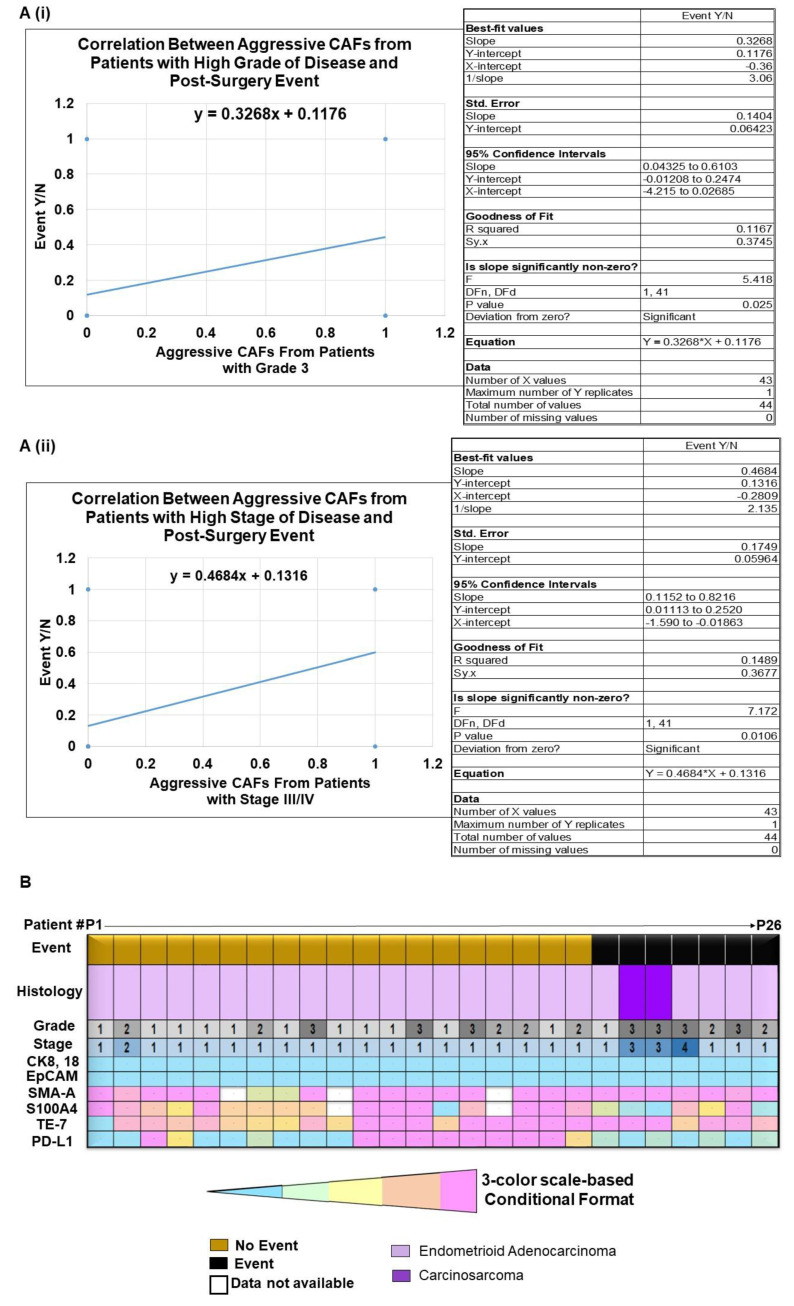

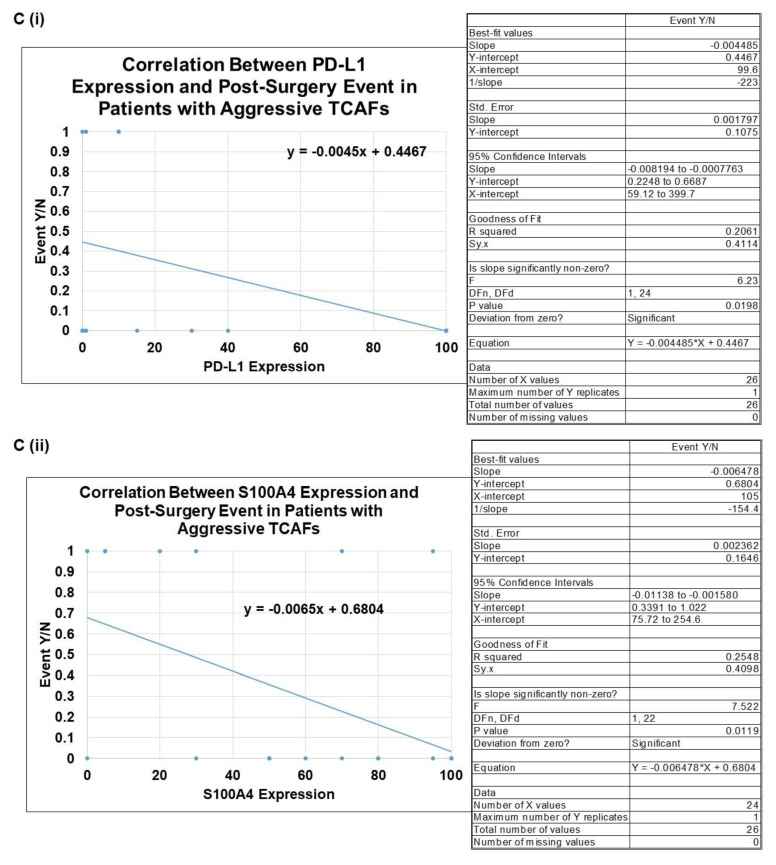
Pearson correlation between aggressive primary CAFs from patients with high grade (Grade 3)/high stage (Stage III/IV) of disease as well as the relationship between % of ICC expression of PD-L1 and S100A4 in aggressive TCAFs and a post-surgery event: Trendline showing the Pearson correlation (95% Confidence Intervals) between aggressive CAFs from patients with high grade (Grade 3) disease and a post-Surgery event. *p* = 0.0250 (**A**(**i**)). Trendline showing the Pearson correlation (95% Confidence Intervals) between aggressive CAFs from patients with higher stage (Stage III/IV) disease and a post-Surgery event. *p* = 0.0106 (**A**(**ii**)). *n* = 43 (Created using GraphPad Prism Version 9.4.1) (**A**). A heatmap of ICC % expression of TCAF markers of the aggressive CAFs sorted by events and no-events is presented (**B**). A correlation between % expression PD-L1 in the aggressive TCAFs and events (**C**(**i**)) and a correlation between % expression S100A4 in the aggressive TCAFs and events **(C(ii))** are presented (**C**). We presented the aggressiveness by (present or absent) and the PSE by (PSE recorded by Sept. 2022 designated as “Yes” or No PSE recorded by Sept. 2022 as “No”) to binary states. Several points overlap because the patient data are the same (for example, we have many patients who were both aggressive CAF and high grade, but it only shows one point on the graph because they share the exact same XY coordinate).

To test the clinical relevance of CAFs, we have conducted follow-ups on the outcome data from patients bearing both aggressive and non-aggressive CAFs. We recorded that 7 patients out of the 31 patients bearing aggressive CAFs had PSEs, while zero events were observed among the patients with non-aggressive CAFs. We tested the correlation between a PSE during the follow-up period and the presence of aggressive CAFs in patients with both high grades and stages of the disease. Figure 4A presents the positive correlation between aggressive-CAF-bearing patients with a high grade (Grade 3) or stage (Stage III/IV) of the disease and post-surgery events. Trendline showed the Pearson correlation (95% Confidence Intervals) between aggressive CAFs obtained from patients with high-grade (Grade 3) disease and a PSE. *p* = 0.0250 (Figure 4A(i)). Trendline showed the Pearson correlation (95% Confidence Intervals) between aggressive CAFs from patients with high-stage (Stage III/IV) disease and a PSE. *p* = 0.0106 (Figure 4A(ii)). The data present a positive correlation indicating a functional relationship between the aggressiveness of the CAFs at higher grades and stages of the disease and the occurence PSEs in endometrial cancers. Figure 4B presents a heatmap of the ICC % expression of the TCAF markers sorted by events and no-events for a total of 26 patients (Event = 7, Non-Event = 19). Interestingly, both patients with carcinosarcomas bearing a high grade and stage of the disease had events. Since we had a distribution pattern for PD-L1 and S100A4 expression in TCAFs with No-events (*n* = 19) and With-event (*n* = 7), we tested the correlation between the % expression PD-L1 (C(i)) and S100A4 (C(ii)) in TCAFs and events separately (Figure 4C). Trendline demonstrated the Pearson correlation between a post-surgery event and the PD-L1 expression levels as determined by ICC in aggressive TCAFs from 26 patients (*p* = 0.0198, *n* = 26), as created using GraphPad Prism Version 9.4.0. Similarly, Trendline demonstrated the Pearson correlation between a post-surgery event and the S100A4 expression levels as obtained by ICC (right panel) in aggressive TCAFs from 24 patients (*p* = 0.0119, *n* = 24), as created using GraphPad Prism Version 9.4.0. Table 3 presents the pathological parameters, the treatment history, and the details of the post-surgery events in seven patients bearing aggressive CAFs. Table 3 shows the central theme of the study: that, despite different pathological and genomic parameters of the tumors in patients, PSE was recorded in all cases bearing aggressive CAFs. By designating the aggressiveness of the CAFs from each patient’s tumor sample, we tested the clinical relevance of the aggressiveness of CAFs and demonstrated that the occurrence of PSEs in patients with a high grade and stage of the disease is directly correlated with the aggressive nature of CAFs in endometrial cancers.

**Figure 5:**
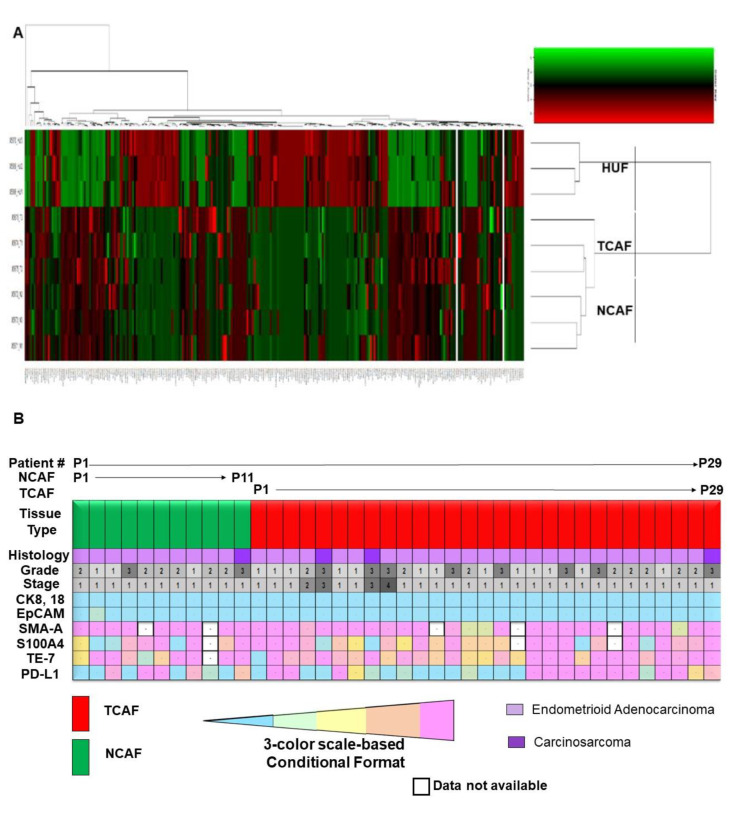
A comparison of NCAF with TCAF in terms of the alterations in genes and expression of protein markers: A comprehensive cancer panel report on all exon coverage on 409 genes involved in some of the most common known cancers shows the alteration of genes in aggressive TCAFs and NCAFs from the same patient compared to HUF cells (**A**). The pathway-based gene selection profiled the mutational spectrum in known cancer driver genes and drug targets along with signaling cascades, apoptosis genes, DNA repair genes, transcription regulators, inflammatory response genes, and growth factor genes. The panel covered both somatic and germline mutations. Heatmaps depicting the genetic alterations identified in NCAF, TCAF, or HUF samples are presented. The coding regions of 409 cancer-related genes are sequenced. Variants with a significant allele frequency difference between sample groups (*n* = 3) are summarized. Numbers of significantly altered variants of those genes are plotted. A 3-color scale-based conditionally formatted expression (% of expression by ICC) of CAF markers in aggressive TCAFs, and NCAFs from 29 representative patients sorted on the basis of CAFs with corresponding histologies, grades/stages (as presented with color codes, and numbers) is presented in a heatmap (**B**).

Figure 5A shows the heatmap for the alterations of genes from one of the aggressive CAFs between TCAF and NCAF compared to those of HUF cells. NCAF and TCAF were strikingly different in the alteration of genes from the HUF cells’ heatmap expression, while the patterns of the alterations of genes were comparable between NCAFs and TCAFs. We report a comprehensive cancer panel on all exon coverage of 409 genes (most commonly altered in neoplasms) in aggressive TCAFs and NCAFs from the same patient (Figure 5A). The panel covered both somatic and germline mutations of genes.

Heatmaps depicting the genetic alterations identified in NCAF, TCAF, or HUF samples are presented. The coding regions of 409 cancer-related genes are sequenced. The variants with a significant allele frequency difference among the sample groups (*n* = 3) are summarized. The numbers of significantly altered variants of those genes are plotted. The data show a clear segregation of HUF expressions from both NCAFs and TCAFs, while the expression pattern of NCAF and TCAF pairs appears comparable. Interestingly, we observed a comparable pattern of the expression of CAF markers between NCAF and TCAF pairs, with a few exceptions, such as S100A4 and PD-L1 in some patients. In line with the above data, a heatmap generated out of the % expression as obtained by ICC in NCAF and TCAF (Figure 5B) demonstrated (1) a uniformity of the negative expression of the epithelial markers CK 8,18,19 and EpCAM in all NCAFs and TCAFs, irrespective of the stage, grade, and histology of the tumors, (2) a uniformity of the high expression of the CAF markers SMA and TE-7 in all NCAFs and TCAFs, irrespective of the stage, grade, and histology of the tumors, and (3) a differential expression of S100A4 and PD-L1 in NCAFs and TCAFs, irrespective of the stage, grade, and histology of the tumors. We studied the clinical relevance of the characterization of CAFs into aggressive and non-aggressive forms by evaluating the aggressiveness of the CAFs and PSE in the EMR. PSE was recorded in 23% of patients with aggressive CAFs, irrespective of the grade, stage, and genomic alterations. No PSE was recorded in our patients bearing non-aggressive CAFs. We observed an inverse correlation between PSE and S100A4 expression (the same is also true for the PD-L1 expression) (Figure 4C). In line with the above results, we observed (1) a higher expression of PD-L1 and S100A4 as determined by both qRT-PCR and WB (Figure 1) in the non-aggressive-TCAF-bearing patient, and (2) a lower expression of PD-L1 and S100A4 as determined by both flow cytometry and WB (Figure 2D) in the aggressive-TCAF-bearing patient.

### 2.4. Growth Resistance Property of Aggressive Endometrial CAFs

**Figure 6:**
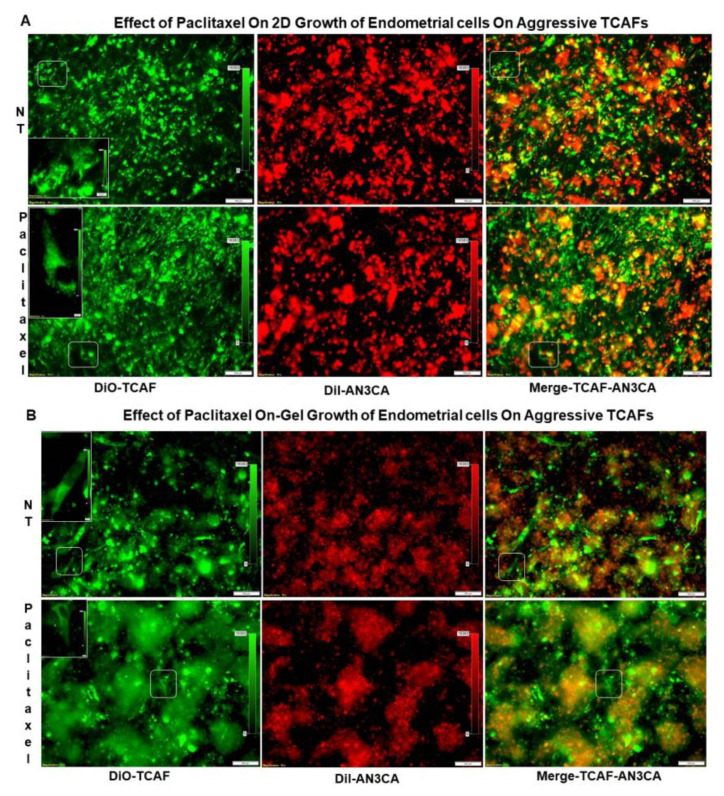

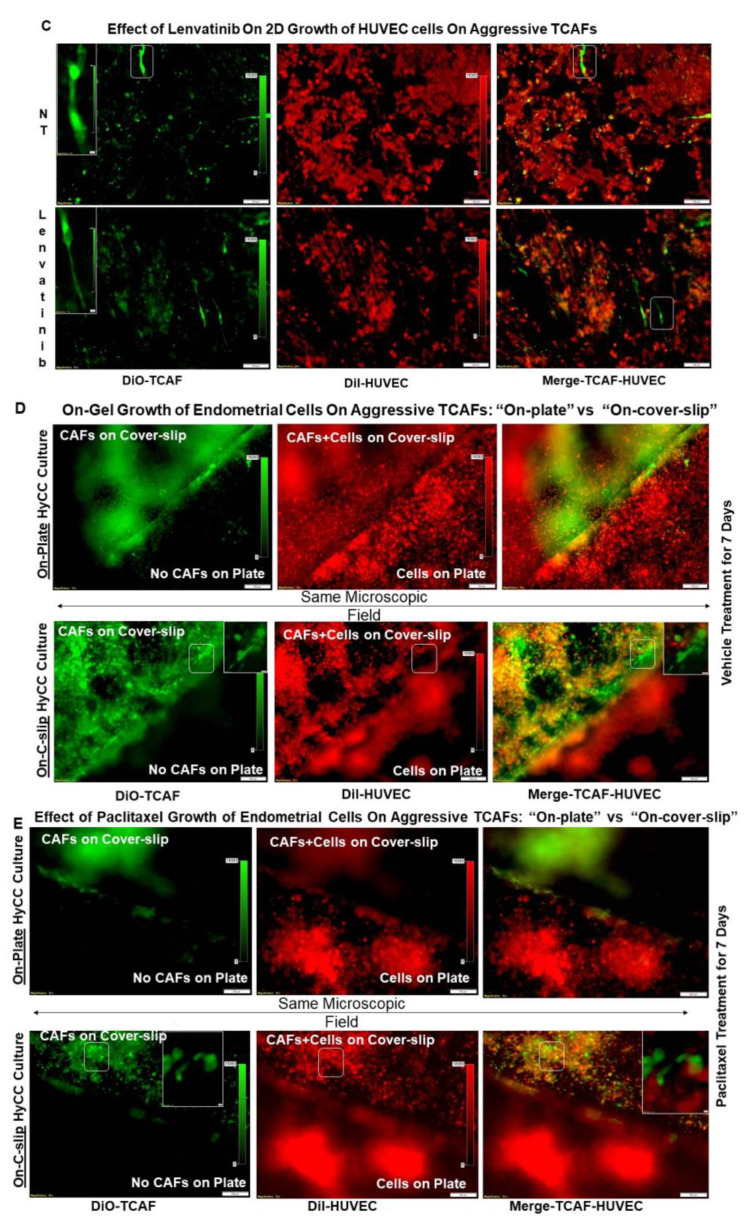
Effect of aggressive CAFs from a patient with PSE on the paclitaxel-mediated inhibition of clonogenic growth of endometrial cancer cells and on the lenvatinib-mediated inhibition of HUVEC growth in hybrid-co-cultures using different formats: Primary culture of aggressive CAFs (passage # 2) from a patient with PSE was stained with DiO and plated on matrigel 24 h before DiI stained AN3CA endometrial cells were added in the 3D On-Top clonogenic cultures with or without 20 nM paclitaxel. Parallel experiments were run without CAFs. Pictures were taken at 20X/40X dry-objective of Olympus BX43 Microscope using cellSens 1.18 LIFE SCIENCE IMAGING SOFTWARE (OLYMPUS CORPORATION). Photomicrographs show the information stamps for magnifications and the scale bars. Growth (2D) of DiI-stained AN3CA endometrial cells in HyCC without coverslips on DiO-stained aggressive endometrial TCAFs with PSE in the presence and absence of paclitaxel for 7 days (**A**), on-gel growth of DiI-stained AN3CA endometrial cells in HyCC without coverslips on DiO-stained aggressive endometrial TCAFs with PSE in the presence and absence of paclitaxel for 7 days (**B**), no-gel-growth of DiI-stained HUVEC (passage # 3) in HyCC on DiO-stained aggressive endometrial TCAFs with PSE in the presence and absence of lenvatinib for 7 days (**C**), and “On-plate” (upper panel) versus “On-C-slip” (lower panel) matrigel On-Top growth in HyCC in the absence (**D**) or presence of paclitaxel (**E**) for 7 days are presented as marked in the photomicrographs. Bar represents 100 micron (µM).

Searching for the mechanistic explanation for the clinical relevance of patient-derived aggressive CAFs in endometrial cancers, we tested the role of aggressive CAFs in resisting the effects of the chemotherapy drug paclitaxel on endometrial tumor cells. Figure 6A presents the effects of the aggressive CAFs cultured from the tumor sample of a patient with a post-surgery event in resisting paclitaxel-mediated growth of endometrial tumor cells following seven days of treatment. The effect of paclitaxel on the clonogenic growth of endometrial tumor cells plated on the aggressive TCAFs derived from patients with PSEs using a HyCC was tested, and the results indicate that TCAF resisted the inhibitory effect of paclitaxel on the 3D clonogenic growth of AN3CA cells (Figure 6B). Neither the on-plate nor on-Top 3D matrigel clonogenic growth of the tumor cells was inhibited following treatment with paclitaxel in the presence of representative aggressive TCAFs at 48 h. At similar times, the same aggressive CAFs also blocked the effect of lenvatinib on HUVEC cells (Figure 6C). The parallel experiments showed the inhibitory effects of the same doses of paclitaxel and lenvatinib on the 3D growth of the test cells. Paclitaxel inhibited the growth of AN3CA. Thus, aggressive TCAFs from patients with PSEs resisted the effects of paclitaxel and lenvatinib on growth in a HyCC. Since paclitaxel failed to inhibit the growth of endometrial tumor cells in the presence of the aggressive CAFs, we tested the basic mode of action of the CAFs by physically isolating the 2 HyCC culture formats of “On-C-slip” HyCC and “On-Plate” HyCC (Figure 6D,E). Diagrammatic representation of the formats of “On-C-slip” HyCC and “On-Plate” HyCC are shown in Figure S1. “On-C-slip” HyCC included cover-slips pre-coated with DiO-stained CAFs on which DiI-stained AN3CA cells were plated. “On-Plate” HyCC did not include pre-coated (with DiO-stained CAFs) cover-slips and consisted only of DiI-stained AN3CA cells plated on the plate. Interestingly, we observed no change in the growth of the endometrial cells between the two formats following the application of paclitaxel, indicating that the growth-inhibition-resisting effect of CAFs does not require direct cell contact and can be mediated via secretomic signals in the media covering the cells in both the formats. Merged pictures of the DiO-stained aggressive CAF and DiI-stained AN3CA of the “On-C-slip” HyCC and “On-Plate” HyCC of the vehicle-treated cells from the same microscopic field showed on dramatic difference in the patterns of cell growth (Figure 6D; upper right panel vs. lower right panel pictures), which was also comparable to the paclitaxel-treated HyCC (Figure 6E; upper right panel vs. lower right panel pictures).

## 3. Discussion

We explored the functional association between aggressive CAFs and resistance to the growth-inhibitory effect of chemo/targeted drugs in endometrial cancers. CAFs are the most abundant stromal cells and play a significant contextual role in shaping tumor initiation, disease progression on therapy, and metastasis. Understanding the function of CAFs, the most influentially abundant stromal cells with a significant contextual role in the progression and metastasis of several solid tumors [6,8], has encouraged CAF-directed clinical trials as part of a next-generation cancer drug design and discovery innovation strategy. CAFs have therefore begun to emerge as a part of future cancer management.

Contrary to the standard system of isolating/growing CAFs based on enzyme digestion, our system is not artificial, as we derived the CAFs from surgically resected tumors and tumor-adjacent normal tissues following pathological grossing in each case. Considering the diversity of the tumor sample received in each case, their grossing pattern, the histology, and the stage/grade of the tumor, we designed a unique way to set up the primary CAF culture from the feeder layer, NOT from the standard enzyme digested model which can give the number of cell counts. To the best of our knowledge, this is the first report of a non-enzymatic isolation of CAFs and their clinical relevance in endometrial cancers. We believe that this novel way of using a natural method of isolating the CAFs has empowered us to identify two different populations of CAFs. Keeping in mind the nature of the heterogeneity of samples, we have performed “viability of the tissues, as well as tumor/stroma ratio of each and every sample”.

As we were aware of the intratumoral heterogeneity of the CAF population within the tumor sample, we used the feeder layer from the entire tissue sample provided to us to set up the primary culture. It is possible that the CAF population we cultured may have more than one subpopulation of CAFs, since the tumor sample(s) we received may well represent a heterogeneous TME (CAF) ecosystem, as reported in PDACs. Hence, our CAFs from a single patient could be a heterogeneous mixture (of different CAF subpopulations), as heterogeneous as it could possibly be in the original tumor sample from that particular patient. Indeed, our data demonstrated that some cells of the CAFs from the same patient bear multiple nuclei, while other CAFs in the same population are mono-nuclear in the same passage (Figure 3A–C).

We identified and characterized CAFs in the context of their clinical relevance in endometrial cancers to demonstrate that a direct correlation exists between the presence of aggressive CAFs and PSE in patients with endometrial cancers. In establishing a patient-derived primary culture of endometrial CAFs, we tested every passage of the NCAFs and TCAFs as per their availability in the primary culture. We considered both the properties of a primary culture of the CAFs, the time of establishment, and the number of passages before the cells show the senescence markers to designate them as aggressive and non-aggressive CAFs. No uniform CAF marker is expressed in the same pattern across all cancers [6]. We demonstrated that the endometrial CAF markers in our cohort bear a characteristic expression pattern in terms of mRNA (Figure 1), protein (Figure 2), and their subcellular localization (Figure 3). Although we observed a comparable % expression of markers between NCAFs and TCAFs as obtained by parallel qRT-PCR, flow cytometry, and ICC, we observed a trend in the classification of CAFs based on the differential expression of S100A4 and PD-L1. Interestingly, we observed a significant positive correlation between the expressions of S100A4 and PD-L1 in CAFs (*p* = 0.0373) (Figure 3D). However, the clinical significance of such observation remains unclear. We observed that NCAFs are lesser in number than TCAFs in our cohort. Delving into the reason for this observation (that NCAFs are lesser in number than TCAFs) in our cohort, we found that fewer TAN tissues were received compared to the tumor tissues obtained. Furthermore, sometimes we received samples from the Fallopian tubes as the TAN samples, and no NCAFs could be established from those samples. This fact indicates that NCAFs are fundamentally different from the fibroblasts of the distant normal tissues of a different organ (more so in the case when the disease is of low grade and stage and is strictly localized). In support of the above logic, we have also observed no difference in the sequencing data for alterations in genes between NACFs and TCAFs of one of our patients bearing aggressive NCAFs and TCAFs as compared to HUF cells as presented in the heatmap (Figure 5). We used HUF only as an internal technical control, since the background data on endometrial CAF are very limited in the literature.

Additionally, we did not find an overall difference in the expression of markers between NACFs and TCAFs in ICC (Figure 5B).

To test whether the aggressiveness of the patient-derived CAF can be regarded as a companion marker with a high grade/stage of the disease for a PSE, we tested the correlation between events and the high-grade/stage patients bearing aggressive CAFs. Interestingly, all 31 patients of the “aggressive CAF category” had TCAFs. Our study has limitations. Our cohort consists of 53 patients with endometrial cancers over a period of 5 years, with most of the patients bearing stage I and grade 1 disease, which naturally caused a skewness towards low grades/stages in the population of patients. Our CTC data provided very limited information, as we did not receive a longitudinal CTC. Since non-aggressive CAFs did not grow beyond passages 2–3 over a longer period (more than a month), the yields of non-aggressive CAFs were practically too low for any experiments other than the testing of markers by ICC. Hence, we could not compare the aggressive and non-aggressive CAFs for the HyCC. One of the limitations of our study is that, due to the nature of the growth patterns of the endometrial sample-derived primary CAFs, the number of samples for some experiments was low for certain categories of experiments. Using single-cell sequencing of tissue-purified CAFs, a subpopulation of aggressive CAFs with a specific gene expression pattern has been defined in several works of the organ-type cancers whose CAFs are already characterized, such as PDAC, melanoma, colorectal, breast, lung, and ovarian cancers. In all of these organ types, the CAF was found to have clinical relevance. Unfortunately, the work has not progressed to that level in the case of endometrial cancers yet. Since the markers of the CAF subpopulations are organ-type specific, we will need more information before we start to tease out subpopulations of CAFs in endometrial cancers per se. In this study, we first sought to find the clinical relevance of the CAFs in endometrial cancers.

We show a positive correlation between patients with grade 3 disease bearing aggressive CAFs and a PSE (*p* = 0.0106), as well as between patients with stage 3/4 disease bearing aggressive CAFs and a PSE (*p* = 0.0017). Considering the fact that the probability of PSE is a function of time in patients and is closely associated with disease progression, we observed PSE in most of the earlier patients in our cohort. It should be noted that the PSEs currently observed in 7 out of 31 patients bearing aggressive CAFs may increase over time, and we may observe a greater number of patients with PSEs within the cohort of aggressive CAFs. Our study is the first to report a correlation between the post-surgery event and the aggressive nature of CAFs in endometrial cancers and provides an undeniable reason to study the in-depth mechanism of CAF function in the context of tumor cells and the rest of the stromal cells of a TME, immune cells, and angiogenic cells. We chose to test the effects of paclitaxel and lenvatinib because these are among the drugs which are clinically used in treating endometrial cancers [11,12]. We provide the first experimental evidence of the mode in which the role of CAF is proved in resisting the growth-inhibitory effect of both paclitaxel and lenvatinib via secretomic signals (Figure 6). Ascertaining direct experimental proof of the role of CAFs in developing resistance to anti-tumor drugs will provide an opportunity to investigate new drugs for counteracting CAF-mediated treatment resistance and thus normalizing TME in aggressive endometrial cancers.

Thus, our tumor cell-on-CAF ex vivo HyCC model provides a unique opportunity for the personalized testing of anti-tumor drugs, positively predicting the development of future resistance well before it is clinically encountered in patients. Including anti-stromal therapy to normalize the TME will broaden drug design and target the TME. Our data prove that the horizon ought to be widened to include the molecular targets contributed by the oncologic signals from CAFs. As we progress on the roadmap for effective cancer management in the coming decade, we are optimistic about *a CAF-inclusive target*.

Currently, the management of the disease in endometrial cancers is targeted at the tumor and the immune compartment of the TME [7,13,14]. Our data can initiate a different perspective to include CAF-directed therapy, especially in the high-risk patients undergoing surgery with high grade and stage of the diseases. Together, our study sheds light on the development of resistant conditions in the presence of aggressive CAFs and suggests a new therapeutics potential for CAF-targeted therapy to enhance the response to cancer treatment and outcome.

## 4. Methods and Materials

### 4.1. Tissue Collection at the Time of Surgery

All experimental protocols were approved by the institutional and/or licensing committee(s). Informed consent (IRB approved: Protocol Number Study: 2017.053-100399_ExVivo001) was obtained from 53 patients and/or their legal guardian(s). The resected tumor (T) and tumor-adjacent normal (TAN) tissues were collected during surgery in designated collection media as per the guidelines and relevant regulations and provided by the pathologist, depending upon the availability of the tissue on a case-to-case basis. We included samples from consecutive consented patients with endometrial tumors at any stage/grade of the disease undergoing surgery (Table 1) with or without pre-treatment/history of any previous carcinoma.

### 4.2. Cell Lines and Reagents

Human uterine fibroblasts (HUF; Primary Uterine Fibroblasts, Cat # PCS-460-010), HUVEC cells (cat # PCS-100-013), endometrial cells (RL-95-2 and AN3CA), MCF7 cells, and NCI-H441 cells were procured from ATCC (USA) and were cultured according to the standard cell culture procedures as per ATCC (USA). MCF-7 cells were cultured in atmospheric 95% air, 5% CO_2_. The complete medium consisted of the base medium for this cell line (ATCC-formulated Eagle’s Minimum Essential Medium, Catalog No. 30-2003). To make the complete growth medium, we added 0.01 mg/mL of human recombinant insulin and fetal bovine serum to a final concentration of 10% and 1% pen/strep (Temperature 37 °C). RL-95-2 cells were cultured at 37 °C and 5% CO_2_. The complete medium for this cell consisted of the base medium (ATCC-formulated DMEM: F12 Medium Catalog No. 30-2006). To make the complete growth medium, we add 0.005 mg/mL of insulin fetal bovine serum to a final concentration of 10% and 1% pen/strep. The subculturing procedure included the removing the growth medium, rinsing with 0.25% trypsin, 0.03% EDTA solution, and allowing the flask to sit at room temperature (or at 37 °C) until the cells detached. Fresh culture medium was added into new culture flasks in a subcultivation ratio of 1:2 to 1:5. AN3CA cells were cultured in atmospheric 95% air, 5% CO_2_. The complete medium consisted of the base medium for this cell line (ATCC-formulated Eagle’s Minimum Essential Medium, Catalog No. 30-2003). To make the complete growth medium, we added a fetal bovine serum to a final concentration of 10% and 1% pen/strep (a subcultivation ratio of 1:3 to 1:6). The NCI-H441 cells’ complete medium consisted of the base medium for this cell line (ATCC-formulated RPMI-1640 Medium, ATCC 30-2001). To make the complete growth medium, we added fetal bovine serum (ATCC 30-2020) to a final concentration of 10% and 1% pen/strep (a subcultivation ratio of 1:3 to 1:8). HUVEC cells were cultured in a vascular cell basal medium (ATCC PCS-100-030) supplemented with endothelial cell growth kit BBE (ATCC PCS-100-040) at 37 °C and 5% CO_2_. The complete medium for HUF cells (Primary Uterine Fibroblasts; Normal, Human (ATCC PCS-460-010) consisted of the components of the growth kit with the Fibroblast Basal Medium-Low Serum (ATCC PCS-201-041), which were added to the basal medium (ATCC PCS-201-030). The Fibroblast Growth Kit-Low Serum components are rh FGF b, 0.5 mL (final concentration 5 ng/mL), L-glutamine, 18.75 mL (final concentration 7.5 mM), Ascorbic acid, 0.5 mL (final concentration 50 μg/mL), Hydrocortisone Hemisuccinate, 0.5 mL (final concentration 1 μg/mL), rh Insulin, 0.5 mL (final concentration 5 μg/mL), and Fetal Bovine Serum, 10.0 mL (final concentration 2%) at 37 °C, and 5% CO_2_. Cells were seeded in a new flask during subculturing at a density of 2500 to 5000 cells per cm^2^. We bought HUF cells from ATCC (ATCC PCS-460-010). These primary uterine fibroblast cells are bipolar, refractile, and adherent spindle-shaped, were isolated from the wall of a donor’s uterus, and were obtained from the non-decidualized uterus via hysterectomy. We have also used primary fibroblasts from the tumor-adjacent normal tissue of the patient as provided to us by the pathologist after grossing following the surgical resection.

Other cell lines for qRT-PCR were procured from ATCC. Antibodies for ICC were bought from Cell Marque (Rocklin, CA, USA), NOVUS (Centennial, CO, USA), Abcam (Waltham, MA, USA), Agilent-Dako (Santa Clara, CA, USA), and Cell Signaling (Danvers, MA, USA). All cells were used within 7–8 passages and tested negative for mycoplasma.

### 4.3. Patient-Derived Primary Culture of Endometrial CAFs

The primary culture of CAFs (TCAF and NCAF) from the endometrial T and TAN samples was set up from the feeder layer. The initial seeding of cells was cultured in media containing DMEM/F-12 + Glutamax. The primary culture of CAFs (TCAF and NCAF) was set up from the resected endometrial tumors and tumor-adjacent normal tissue samples. Since the culture was initiated from a feeder layer, the entire process was independent of any enzymatic digestion of the tissue. The initial seeding of cells was cultured in media containing DMEM/F-12 + Glutamax. Once the fibroblast cells grew in passage zero, they were passaged thereof by differential trypsinization (trypsin-sensitive and trypsin-resistant cells). We monitored the purity and the extent of the epithelial cell contamination of the cultures by testing (1) the negative expression of non-CAF markers, including epithelial cell markers CK 8,18,19, and EpCAM, leucocyte common antigen CD45, and endothelial cell marker CD31, and (2) the positive expression of fibroblast/CAF markers, including SMAalpha, S100A4, CD90, FAP, CD155, TE-7, PDGFRA, and FGFR. Expression of the stem cell marker CD44 and the immune checkpoint marker PD-L1 are also monitored. The expression of fibroblast markers was monitored throughout each of the following passages until the late passages tested positive for beta-galactosidase stain. We employed qRT-PCR, Western blot, flow cytometry, and ICC to test the expression of the above-mentioned markers (mRNAs and proteins). The purity and the extent of epithelial cell contamination of the cultures were monitored by testing the expression of mRNA by qRT-PCR as well as the protein expression by flow and ICC. Acknowledging the fact of CAF heterogeneity and the fact that very limited data have been published in the literature on the endometrial CAFs, we examined three categories of CAF markers in our study, (1) negative markers, (2) positive markers, and (3) other associated immune markers and stem cell markers in paired NCAF and TCAF samples. We have also presented a simultaneous readout by using two testing methods for multiple markers (positive) in the paired samples for clarity.

A passage of the primary culture of CAF is qualified by (1) the negative expression of non-CAF markers, including epithelial cell markers CK 8,18,19, and EpCAM, leucocyte common antigen CD45, and endothelial cells marker CD31, and (2) the positive expression of fibroblast/CAF markers, including SMAalpha, S100A4, CD90, FAP, CD155, TE-7, PDGFRA, and FGFR. The expression of stem cell marker CD44 and immune checkpoint marker PD-L1 were also monitored. The expression of fibroblast markers was monitored throughout each passage. The first 3 passages are designated as early, followed by mid and late passages. Depending upon the viability and expression of the markers, the late passage CAFs have been tested for senescence (beta-galactosidase assays), as presented in Figure 3E.

### 4.4. Comprehensive Cancer Panel for Aggressive CAF Pair and HUF

We obtained a Comprehensive Cancer Panel (CCP) for an aggressive NCAF-TCAF pair and compared it with HUF cells (a ‘commercially available DNA sequencing CCP’ from PrimBio Research Institute, Exton, PA, USA). The CCP (DNA sequencing panel) consisted of the most comprehensive cancer panel available, with all exon coverage on the 409 genes involved in the most common known cancers. The workflow consisted of the construction of the library using the Ampliseq 2.0 Library Kit (Cat. no. 4475345), preparation of the template, sequencing (An Ion S5 sequencer using an Ion Torrent Amplieseq CCP DNA run plan), and data analysis (Ion Torrent Browser Plugin for VCF generation and data uploaded to Ion Reporter). Approximately 50 pM of pooled libraries were used for templating using the Life Technologies Ion Chef S5 kit (Cat# 4488377) and the manufacturer’s recommended protocol.

### 4.5. Expression of mRNA for CAF Markers by qRT-PCR

As mentioned earlier, the expressions of CAF markers were tested by qRT-PCR. RNA extraction and qRT-PCR were performed as mentioned elsewhere [15]. In short, RNA extraction was performed using the Qiagen RNEasy MiniKit and Qiashredder Kit according to the manufacturer’s protocol. RNA was extracted from lysed cell pellets (Qiashredder system) and converted to cDNA using iScript Reverse Transcription Supermix. qRT-PCR was performed using the Roche LightCycler96 platform. Appropriate primers (Integrated DNA Technologies, Coralville, IA, USA) for each gene of interest were mixed with Roche FastStart Essential DNA Green Master Mix and run in triplicate. FastStart Essential DNA Green Master (Roche, Basel, Switzerland) was used for product detection (Relative Ratio of the gene of interest to GAPDH) using the Roche LightCycler 96 Software version 1.1. The list of primers used is presented in Table 4.

### 4.6. Expression of Protein Markers of CAF by Flow-Cytometer and Western Blot

Flow cytometry was performed using SMA-FITC, FAP-PE, S100A4-PerCP, EpCAM-APC, CD31-FITC, CD155-PE, CD90 PE-Vio615, and PD-L1- APC. For flow cytometry, cells were trypsin released and rinsed in FACS Buffer (phenol red-free RPMI with 1% FBS). Cells were stained for 15 min with cell surface antibodies (CD31 Miltenyi, CD155 Miltenyi, CD90 Miltenyi, FAP R&D systems, PD-L1 Miltyeni) or corresponding isotype control antibodies (Miltenyi). Cells were fixed using the kit from Miltenyi for 30 min, followed by re-suspension in a permeabilization buffer from the same kit. Cells were stained for intracellular antibodies (SMA and S100A4, both from Novus biologicals) for 30 min. Stained cells were run on a BD Accuri C6 flow cytometer and analyzed using FCS express (DeNovo software, 7.16.0047). TCAFs and NCAFs from all patients with established CAFs at every passage of the primary culture were tested for the expression of markers to confirm specificity and aggressiveness. The list of antibody conjugates used in the study is presented in Table 4. The expression of a few CAF markers (qRT-PCR, flow cytometry, and Western blot) in representative aggressive and non-aggressive CAFs, one each, was shown (Figure 1 and Figure 2).

### 4.7. Cellular Localization of CAF Markers by ICC

For ICC, CAFs were cultured on coverslips. Both NCAFs and TCAFs from each passage were stained for EpCAM, CK 8,18, SMAalpha, S100A4, TE-7, and PD-L1 to confirm the specificity and aggressive nature of the CAFs. ICC for protein was first validated and evaluated by a pathologist and then was run with corresponding positive and negative controls. Endometrial tumor cells (RL-95-2 and AN3CA) were used as the positive control for EpCAM and CK 8,18 and as the negative control for SMAalpha, S100A4, and TE-7. HUF was used as the positive control for SMAalpha, S100A4, TE-7, and the negative control for EpCAM and CK 8,18. HUVEC cells were used as the negative control for EpCAM, CK 8,18, SMAalpha, S100A4, and TE-7 and as the positive control for CD31. NCI-H441 cells were used as the positive control for PD-L1, and MCF7 cells were used as the negative control for PD-L1. Hematoxylin was used as the counterstain. For ICC, pictures were taken at 20× and 40× dry-objectives of an Olympus BX43 Microscope using cellSens 1.18 LIFE SCIENCE IMAGING SOFTWARE (OLYMPUS CORPORATION). The list of antibodies used is presented in Table 4.

### 4.8. Categorization of the Aggressiveness of CAFs

CAFs were categorized according to their pattern of growth. The CAFs from each patient’s tumor sample were designated as either aggressive or non-aggressive CAFs based on two criteria of aggressiveness: first, the time required for establishing the CAF culture in weeks, and second, the number of passages the cultured CAFs were passaged before becoming positive for the senescence stain. An aggressive CAF is defined by an early establishment (within 4–7 weeks) and a higher passage number (>3 passages) of the primary culture. A non-aggressive CAF is defined by a late establishment (more than 4–7 weeks) and a lower passage number (<3 passages) of the primary culture. Each passage of the CAFs was tested for the expression of the negative and positive markers as well as for the senescence markers.

### 4.9. Post-Surgery Events in Endometrial Patients

To test the clinical relevance of the aggressiveness of the CAFs derived from the tumor samples of patients with a high grade/stage of the disease, we tested the correlation between the occurrence of a PSE and the high-grade/stage of the disease in patients bearing aggressive CAFs. The occurrence of PSEs in endometrial patients bearing both aggressive and non-aggressive CAFs was obtained from the EMR in accordance with the IRB approval of the Avera Cancer Institute. Clinical events during the post-surgery follow-up period were recorded along with the treatment(s) received by the patient. The PSEs included (1) the metastatic recurrence of the disease as evidenced by a CT scan or biopsy following vaginal spotting/bleeding, (2) worsening symptoms with a moderate volume of ascites and peritoneal nodularity, (3) death following hematuria and worsening peripheral edema.

### 4.10. Testing the Effect of Aggressive TCAFs from Patients with Post-Surgery Events on the 3D Clonogenic Growth of Endometrial Tumor Cells

We tested the effect of an aggressive TCAF derived from the tumor sample of a patient who presented a PSE. For example, a patient with lymph node-negative grade 1, stage I disease developed squamous cell carcinoma of the skin. A HyCC was performed using a dual format (96-well plates and 48-well plates) of the patient-derived primary TCAF (Figure S1). The endometrial tumor cells, AN3CA, were used as the tumor cell co-cultured on the TCAF. The growth patterns of the TCAF and tumor cells were also separately compared in the presence or absence of paclitaxel in parallel to the HyCC. TCAFs and tumor cells were characterized by flow cytometry before the set-up of the HyCC culture and after DiI and Di-O stains. The HyCC of the patient-derived primary DiO-stained TCAF was set up with DiI-stained endometrial tumor cells, AN3CA, in the presence or absence of 20 nM of paclitaxel. DiO-stained TCAFs were plated on growth factor-reduced phenol-red free matrigel. After 24 h, DiI-stained tumor cells with or without paclitaxel were plated on the DiO-stained TCAFs, and a 3D-on-Top culture was carried out. Paclitaxel’s effect on the 3D growth of tumor cell colonies was recorded over 7 days in the presence or absence of TCAF. Photomicrographs of 3D colonies were captured using Olympus Fluorescence Microscope and cellSens Dimensions.

### 4.11. Statistical Evaluation

We determined the Pearson’s correlation (95% Confidence Intervals) between the existence of aggressive primary CAFs taken from patients with a high grade (Grade 3)/high stage (Stage III/IV) of disease and the occurrence of a PSE by measuring both the strength and direction of the linear relationship between the two continuous variables.

## Figures and Tables

**Table 1:** Information on patients included in the study.

All Patients	
**Age group**	# Patients (*n* = 53)
Below 55	7
Above 55	46
Pathology Parameters	
**Histology**	
Carcinosarcoma	3
Endometrioid Adenocarcinoma	42
Endometrial Cancer Not Otherwise Specified	1
High-Grade Papillary Serous Carcinoma	1
Mixed High-Grade Carcinoma	3
High-Grade Serous Carcinoma	3
**Stage**	
I	40
II	2
III	8
IV	3
**Grade**	
1	27
2	11
3	15
**Myometrial Invasion %**	
0–25	24
26–50	19
51–75	4
76–100	6
**Lymphovascular Invasion**	
+	13
−	39
Indeterminate	1
**Lymph Node Positivity**	
+	11
−	35
None Submitted	7

**Table 2:** Pathological parameters of endometrial cancer patients bearing aggressive and non-aggressive CAFs.

Patients with Aggressive CAF	
Histology	
Carcinosarcoma	2
Endometrioid Adenocarcinoma	26
Endometrial Cancer Not Otherwise Specified	1
High-Grade Papillary Serous Carcinoma	0
Mixed High-Grade Carcinoma	1
High Grade Serous Carcinoma	1
**Stage**	
I	25
II	1
III	3
IV	2
**Grade**	
1	14
2	8
3	9
**Myometrial Invasion %**	
0–25	11
26–50	14
51–75	2
76–100	4
**Lymphovascular Invasion**	
+	5
−	25
Indeterminate	1
**Lymph Node Positivity**	
+	5
−	23
None Submitted	3
**Patients with Non-Aggressive CAF**	
**Histology**	
Carcinosarcoma	1
Endometrioid Adenocarcinoma	9
Endometrial Cancer Not Otherwise Specified	0
High Grade Papillary Serous Carcinoma	1
Mixed High-Grade Carcinoma	1
High-Grade Serous Carcinoma	1
**Stage**	
I	9
II	0
III	3
IV	1
**Grade**	
1	8
2	1
3	4
**Myometrial Invasion %**	
0–25	7
26–50	3
51–75	1
76–100	2
**Lymphovascular Invasion**	
+	5
−	8
Indeterminate	0
**Lymph Node Positivity**	
+	4
−	5
None Submitted	4

**Table 3:** Pathological parameters, genomic alterations, and treatment details of endometrial cancer patients bearing aggressive CAFs who presented with a post-surgery event.

Characteristics of Patients with Aggressive CAF and Post-Surgery Events							
#	Treatment		Post-Surgery Events			Pathology Parameter(s)	Genomics
	Neo-Adjuvant Treatment	Adjuvant Treatment	Specific Event	Time of Event (Months)	OS (Overall Survival in Months)		
1	None	None	Developed “squamous cell carcinoma of the skin”	25–37	NA	Endometrioid Adenocarcinoma	Not performed
						Grade 1	
						Stage I	
						LVI Negative	
						Lymph Node: Negative	
						Myometrial Invasion: 30%	
						Mismatch Repair Competent	
2	None	Carboplatinum + Paclitaxel x6. Radiation post-chemo.	Vaginal bleeding, biopsy demonstrated recurrent disease.	10	14	Carcino-sarcoma	***PTEN***: p.L186fs Frameshift. Variant Allele Fraction 52.1% ***CTNNB1***: p.S33F Missense variant. Variant Allele Fraction 34.2% ***PTEN***: p.R130G Missense variant. Variant Allele Fraction 22.9% ***ARID1A****:* p.P109fs Frameshift. Variant Allele Fraction 10.4%
						Grade 3	
						Stage III	
						LVI Positive	
						Lymph Node: Positive	
						Myometrial Invasion: 72%	
						Mismatch Repair Competent	
3	None	Carboplatinum + Paclitaxel x6. Radiation post-chemo.	CT scan demonstrated lymph nodes concerning for metastatic disease, biopsy confirmed recurrence.	19	NA	Carcino-sarcoma	***PIK3CA***: p.Q546K Missense Variant. Variant Allele Fraction 68% ***CDKN2A***: p.P81L Missense Variant. Variant Allele Fraction 4% ***TP53***: p.K132R Missense Variant. Variant Allele Fraction 53% ***FBXW7***: p.R505C Missense Variant. Variant Allele Fraction 20% ***KMT2D****:* p.Q2811Sfs*40 Frameshift. Variant Allele Fraction 16% ***SLX4***: p.Y255* Nonsense variant. Variant Allele Fraction 17%
						Grade 3	
						Stage III	
						LVI Negative	
						Lymph Node: Positive	
						Myometrial Invasion: 38%	
						Mismatch Repair Competent	
4	None	Carboplatinum + Paclitaxel x6.	Patient had worsening symptoms and was found to have moderate volume of ascites and peritoneal nodularity. CT scan showed changes concerning for progression.	10	13	Endome-trioid Adeno-carcinoma	***PIK3CA***: p.H1047R Missense variant (exon 20). Variant Allele Fraction 52.8% ***PTEN***: p.E288* Stop gain. Variant Allele Fraction 71% ***TP53***: p.Y234C Missense Variant. Variant Allele Fraction 68.5% ***NF1***: Copy Number Loss ***KHDRBS3-TP63***: Chromosomal rearrangement
						Grade 3	
						Stage IV	
						LVI Negative	
						Lymph Node: None Submitted	
						Myometrial Invasion: 50%	
						Mismatch Repair Competent	
5	None	None	Patient presented with hematuria and worsening peripheral edema. There was concern for recurrent disease but patient passed away before definitive diagnosis.	11	11	Endome-trioid Adeno-carcinoma	Not performed
						Grade 2	
						Stage I	
						LVI Negative	
						Lymph Node: Negative	
						Myometrial Invasion: 44%	
						Mismatch Repair Competent	
6	None	Radiation.	Patient presented with vaginal spotting, biopsy revealed recurrent disease. CT scan demonstrated widespread metastatic recurrence.	27	NA	Endome-trioid Adeno-carcinoma	***PIK3CA*:** p.Q546H Missense variant. Variant Allele Fraction 55% **17q12q21.2** (**Focal**—***ERBB2***): Gain, Copies = 3.5 ***SMARCA4***: p.L410Tfs*90 Frameshift. Variant Allele Fraction 28% ***SPOP***: p.R121Q Missense variant. Variant Allele Fraction 59% ***TP53***: p.J195F Missense variant. Variant Allele Fraction 46% **17p13.3q12** (**whole 17p, part 17q—*TP53***): Loss, Copies = 1.5 **10q23.1q23.32** (**Sub-arm—*PTEN***): Loss, Copies = 1.5
						Grade 3	
						Stage I	
						LVI Negative	
						Lymph Node: Negative	
						Myometrial Invasion: 95%	
						Mismatch Repair Competent	
7	None	Radiation.	Patient had lesion on top of vagina, biopsy revealed recurrent disease.	8	NA	Endome-trioid Adeno-carcinoma	Invitae—Negative
						Grade 2	
						Stage I	
						LVI Negative	
						Lymph Node: Negative	
						Myometrial Invasion: 6%	
						Mismatch Repair Deficient	

**Table 4:** Detailed information on the antibodies and primers used in the study.

Primers for qRT-PCR		
Gene	Primer Sequence (Sequences Listed 5′–3′)	
*ACTA-2/SMA*	F: CGT TAC TAC TGC TGA GCG TGA	
	R: GCC CAT CAG GCA ACT CGT AA	
*CD31*	F: ATT GCA GTG GTT ATC ATC GGA GTG	
	R: CTG GTT GTT GGA GTT CAG AAG TGG	
*CD44*	F: AGC ACT TCA GGA GGT TAC ATC T	
	R: CTT GCC TCT TGG TTG CTG TCT	
*CD45*	F: CTTCAGTGGTCCCATTGTGGTG	
	R: CCACTTTGTTCTCGGCTTCCAG	
*CD90/THY1*	F: GAAGGTCCTCTACTTATCCGCC	
	R: TGATGCCCTCACACTTGACCAG	
*EpCAM*	F: AGC GAG TGA GAA CCT ACT GGA	
	R: CGC GTT GTG ATC TCC TTC TGA	
*FAP-A*	F: GGA AGT GCC TGT TCC AGC AAT G	
	R: TGT CTG CCA GTC TTC CCT GAA G	
*GAPDH*	F: TCA AGG CTG AGA ACG GGA AG	
	R: CGC CCC ACT TGA TTT TGG AG	
*FGFR1*	F: GAC ACC ACC TAC TTC TCC GTC AA	
	R: CAA TAT GGA GCT ACG GGC ATA CG	
*PDGFRA*	F: TGG CAG TAC CCC ATG TCT GAA	
	R: CCA AGA CCG TCA CAA AAA GGC	
*PD-L1*	F: ACC TAC TGG CAT TTG CTG AAC G	
	R: ATA GAC AAT TAG TGC AGC CAG GT	
*S100A4*	F: CAG AAC TAA AGG AGC TGC TGA CC	
	R: CTT GGA AGT CCA CCT CGT TGT C	
**Antibodies for ICC**		
Antibody	Manufacturer	Cat.#
Cytokeratin 8 & 18 (B22.1 & B23.1)	Cell Marque	818M-94
Ep-CAM/Epithelial Specific Antigen (Ber-EP4)	Cell Marque	248M-94
Fibroblasts Antibody (TE-7)	NOVUS	NBP2-50082
Actin, Smooth Muscle (1A4)	Cell Marque	202M-94
Recombinant Anti-S100A4 Antibody	Abcam (Waltham, MA, USA)	ab124805
PD-L1 [Clone 22C3]	Agilent-Dako	M365329-1
PD-L2 (D7U8C)	Cell Signaling	82723
CD31	Cell Signaling	3528
Vimentin (SP20) Rabbit Monoclonal Antibody	Cell Marque	347R-14
**Antibodies for Flow Cytometry**		
Antibody	Manufacturer	Cat.#
CD31-FITC	Miltenyi (Waltham, MA, USA)	130-117-390
CD155-PE	Miltenyi	130-105-846
CD90 PE-Vio615	Miltenyi	130-114-909
S100A4-PerCP	NOVUS	NBP2-36431APCCY7
SMA-FITC	NOVUS	NBP2-34522F
FAP-PE	R&D Systems (McKinley Place NE, Minneapolis, MN, USA)	FAB3715P-025
PD-L1-APC	Miltenyi	130-122-816
EpCAM-APC	Miltenyi	130-133-260

## Data Availability

Not applicable.

## Supplementary Materials

The following supporting information can be downloaded at: https://www.mdpi.com/article/10.3390/ijms24076449/s1.
