# Supplementary material for: Characterization and Clinical Relevance of Endometrial CAFs: Correlation between Post-Surgery Event and Resistance to Drugs
Source: Int J Mol Sci. 2023 Mar 29;24(7):6449. doi: 10.3390/ijms24076449 (PMC10094763; doi:10.3390/ijms24076449)
Supplement: Supplementary file 1 [file ijms-24-06449-s001.zip › ijms-2301529-supplementary.pdf]

Suppliematry Figure S1

On Cover-slip & On-plate Plan of Matrigel On-Top Hybrid Co-culture

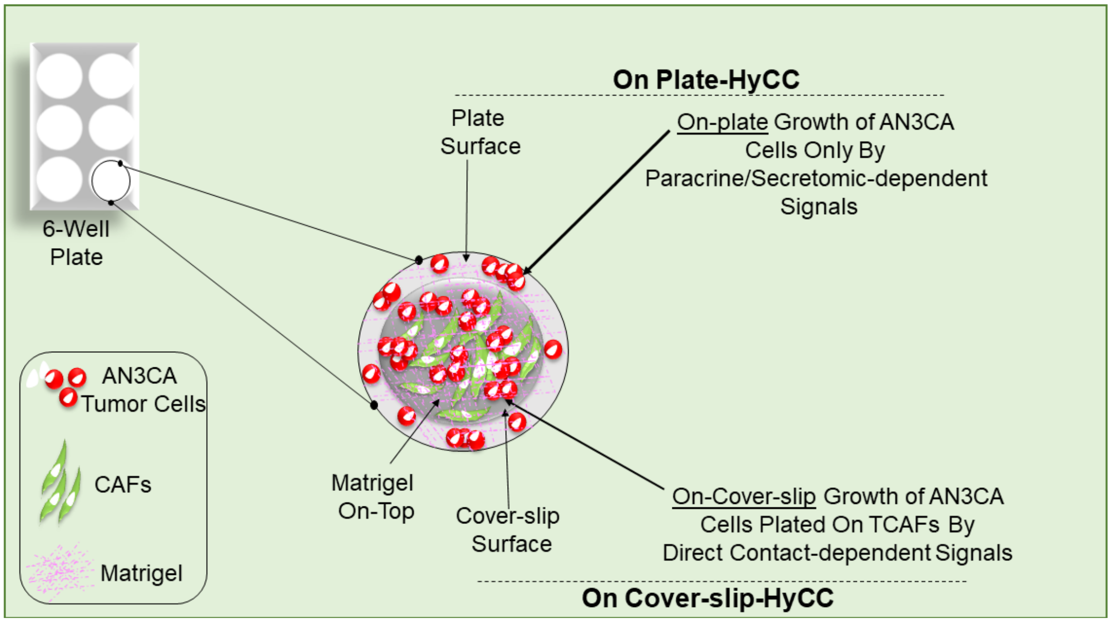

Figure S1:Diagrammatic representation of formats of "On-C-slip" HyCC and "On-Plate" HyCC are shown. "On-C-slip" HyCC comprised coverslips pre-coated with DiO-stained CAFs on which DiI-stained AN3CA cells were plated. While "On-Plate" HyCC comprised no pre-coated coverslips, only DiI-stained AN3CA cells were plated on the plate.

Additional Supplementary Statement:

We received both tumor and tumor-adjacent normal samples from the resected tissue within an hour of the surgery. Depending on the stage, grade, size of the tumor mass, and oncologists' decision, we received the tissue for the grossing (in the pathology department). We received tissues once pathologists retained tissues for the MMR profiling/HRD testing/ future pathology tests as required by the oncologists.

From the pathology department, we received the tissues as follows:

1. Only tumor
2. Tumor and normal endometrium as the tumor-adjacent normal
3. Tumor and Fallopian tube as the tumor-adjacent normal
4. Tumor and normal myometrium as the tumor-adjacent normal
